# A Novel Heteromorphous Convolutional Neural Network for Automated Assessment of Tumors in Colon and Lung Histopathology Images

**DOI:** 10.3390/biomimetics8040370

**Published:** 2023-08-16

**Authors:** Saeed Iqbal, Adnan N. Qureshi, Musaed Alhussein, Khursheed Aurangzeb, Seifedine Kadry

**Affiliations:** 1 Department of Computer Science, Faculty of Information Technology & Computer Science, University of Central Punjab, Lahore 54000, Pakistan; dr.qureshi@ucp.edu.pk; 2 Department of Computer Engineering, College of Computer and Information Sciences, King Saud University, P.O. Box 51178, Riyadh 11543, Saudi Arabia; musaed@ksu.edu.sa (M.A.); kaurangzeb@ksu.edu.sa (K.A.); 3 Department of Applied Data Science, Noroff University College, 4612 Kristiansand, Norway; seifedine.kadry@noroff.no

**Keywords:** bioinspiration, medical image analysis, tumor assessment, convolutional neural network (CNN), heteromorphous deep CNN, histopathology images

## Abstract

The automated assessment of tumors in medical image analysis encounters challenges due to the resemblance of colon and lung tumors to non-mitotic nuclei and their heteromorphic characteristics. An accurate assessment of tumor nuclei presence is crucial for determining tumor aggressiveness and grading. This paper proposes a new method called ColonNet, a heteromorphous convolutional neural network (CNN) with a feature grafting methodology categorically configured for analyzing mitotic nuclei in colon and lung histopathology images. The ColonNet model consists of two stages: first, identifying potential mitotic patches within the histopathological imaging areas, and second, categorizing these patches into squamous cell carcinomas, adenocarcinomas (lung), benign (lung), benign (colon), and adenocarcinomas (colon) based on the model’s guidelines. We develop and employ our deep CNNs, each capturing distinct structural, textural, and morphological properties of tumor nuclei, to construct the heteromorphous deep CNN. The execution of the proposed ColonNet model is analyzed by its comparison with state-of-the-art CNNs. The results demonstrate that our model surpasses others on the test set, achieving an impressive F1 score of 0.96, sensitivity and specificity of 0.95, and an area under the accuracy curve of 0.95. These outcomes underscore our hybrid model’s superior performance, excellent generalization, and accuracy, highlighting its potential as a valuable tool to support pathologists in diagnostic activities.

## 1. Introduction

Cancer, the second biggest cause of death, represents a major worldwide health issue. According to estimates, 609,820 Americans will die from cancer in 2023, which works out to almost 1670 fatalities every day. There may be roughly 153,020 new instances of colorectal cancer (CRC) in the US in 2023, made up of 106,970 tumors in the colon and 46,050 tumors in the rectum [1,2]. There are an enormous amount of cells in the human body, and these cells divide, develop, and multiply. When cells become damaged or reach a certain age, they typically either die naturally or are replaced by healthy counterparts [3]. However, if this replacement procedure does not take place, damaged cells start to multiply and become benign or malignant tumors. While malignant tumors are characterized by their abnormal, fast development and their propensity to infect surrounding tissues, benign tumors are slow-growing masses that do not affect the neighboring cells. Numerous areas of the human body can be affected by cancer cells, but colon and lung disease are among the most frequent, affecting both sexes equally [4].

About 238,340 new cases of lung cancer are anticipated to be diagnosed nationwide in 2023. From this, 117,550 male cases and 120,790 female cases are expected. In terms of new instances, it is predicted that there may be 127,070 new cases of lung cancer overall in 2023. There will be 59,910 new cases of cancer diagnosed in women and 67,160 new cases of cancer diagnosed in men among these [1,4,5,6]. Numerous behavioral variables, such as smoking, being overweight, abusing alcohol, or being exposed to UV radiation, ionizing radiation, or other biological agents, can have an impact on the development of cancer [6]. The co-occurrence of lung and colon cancers, which accounts for around 17% of cases [7], is noteworthy. Smoking, which is added to unhealthy eating habits, has been linked to the emergence of breast and colon cancer, according to research [7]. Early-stage lung and colon cancer sometimes exhibit negligible or no symptoms, delaying identification and confirmation until later stages, when treatment results may be adversely affected [8]. Comprehensive tests including computed tomography (CT-Scans), MRI scans, ultrasound imaging, and tissue samples are required in order to describe lung and colon cancer in its early stages [9].

Individuals who smoke, are overweight, or have a family history of cancer should consider having regular checkups with their doctor. It is important to note that screening procedures can be expensive, making it challenging for people with low incomes to pay them. According to the World Health Organization (WHO), developing low- and middle-income nations account for 70% of cancer-related fatalities. It is essential to help these nations in creating fully functional hospitals with free diagnostic labs for everyone in order to solve this issue. In addition, there is a large time lag and the possibility of divergent medical opinions, especially in the early stages of cancer diagnosis. Collaboration across disciplines within the healthcare industry is necessary to meet these issues. Potential remedies include the use of artificial intelligence techniques like biomedical imaging for illness early detection and emergency healthcare forecasting models [10,11,12].

Our research takes inspiration from nature’s remarkable ability to adapt and optimize biological systems. By leveraging advanced computational techniques, specifically a heteromorphous convolutional neural network (CNN), our study aims to mimic and improve upon the natural mechanisms of tumor assessment in colon and lung histopathology images. The design and optimization of our proposed approach ColonNet draw upon the principles of efficient information processing and pattern recognition. Moreover, the extraction and integration of distinct structural, textural, and morphological properties of tumor nuclei within our model demonstrate an approach inspired by the biological systems found in nature.

Deep learning algorithms are used to analyze data from a variety of sources, such as high-dimensional images, videos, and anatomical representations. These methods make it possible to identify details and traits that may not be evident to the observer from medical images. This is especially helpful for identifying early-stage tumors and discriminating between them. In this work, numerous hybrid systems that include characteristics extracted using a variety of techniques were built. The objective was to blend deep learning features with features that were extracted from other techniques. Each form of cancer is intended to be represented by distinct and strong characteristics that can set it apart from other types of cancer.

Deep learning (DL) for colon and lung cancer diagnosis has lately received a significant amount of interest. The use of histopathological images in automated diagnosis has been the subject of several fruitful investigations. This specific study uses histopathology images alone to automatically find tumors in the colon and lungs. The goal is to divide this dataset (images) into five different categories: squamous cell carcinomas, lung adenocarcinomas, benign lung lesions, benign colon lesions, and colon adenocarcinomas. Through the use of DL methods and superior outcomes, the prognosis of these cancers is intended to be greatly improved. Numerous studies have been conducted using different DL models, and the findings show that the architecture is able to correctly classify various sub-types of colon and lung cancer. The following are some of the major contributions that this work brings:

- In order to precisely separate mitotic nuclei from histopathology images of colon and lung tumor, this work presents a unique hybrid segmentation approach. To improve the robustness and generalization of the classification system, the proposed method combines the distinctive qualities of two different CNN models. The ensemble performs better at reliably recognizing and detecting mitotic nuclei in colon and lung cancer histopathology images by taking advantage of the advantages of each individual model.
- In order to accurately represent the morphological, structural, and textural properties of mitotic cells, two unique CNN models are created based on distinct ideas. Weakly annotated mitotic cells present a number of obstacles, and asymmetric split transform-merge and label optimizer (LO) approaches are developed to solve these issues and reduce false positive and negative errors. A global–local pyramid pattern (GLPP) is utilized for efficient feature extraction and CNN model integration. Additional methods, such as bespoken deep residual network blocks and residual training are used to improve the performance of the models. A custom layer is used to integrate the retrieved features in the center of the CNN model.
- We compare the proposed heteromorphic CNN model with contemporary CNN models such as VGG, ResNet, DenseNet, Inception, and Xception. The evaluation’s goal is to gauge the classification system’s accuracy, and the findings show that the ColonNet model outperforms the current CNN models in terms of performance.

The rest of this article is structured as follows:

Section 2 offers a summary of the related work. In Section 4, we outline the architecture of the ColonNet model. Section 3 describes the datasets that were used in this study, encompassing dataset details, training procedures, and pre-processing. It also includes a thorough analysis of the model’s performance. Section 5 presents the results and comparison with other pre-trained models and discusses their implications. Section 6 explains the contribution to the existing knowledge in the field and potential implications. Lastly, Section 7 discusses the conclusion and potential avenues for future research.

## 2. Background and Related Work

There are two primary causes that make it difficult to segment nuclei: (i) color changes in histopathological images and (ii) variances in morphological features. In the literature, a number of image processing methods have been put out to address nuclei segmentation from histopathology images. These methods include the watershed method [13], the multi-level thresholding examined by the watershed algorithm [14], hybrid segmentation using k-Means clustering and adaptive thresholding [15], the multi-scale and multimarker approaches [16], and the graph-cuts approach [17]. However, because they all rely on parameter-based techniques, these image processing-based algorithms are unable to manage changes in staining and morphological features.

Machine learning (ML) techniques for nucleus segmentation have drawn more and more attention in recent years. Multiple hand-crafted features, including color, texture, the Laplacian of Gaussian response, local binary patterns of the nuclei, the Hough transform, the Histogram of Oriented Gradients (HOGs), and the marker-controlled watershed approach, have been used to train machine learning models [18,19,20]. To analyze and segment nuclei, these models often use supervised or unsupervised learning approaches.

Traditional methodologies, in particular unsupervised learning approaches, have difficulties in nucleus segmentation since they rely heavily on feature engineering. These techniques frequently result in under-segmented nuclear areas when there is noticeable color and textural diversity [21]. It can be difficult to manually recognize and extract useful characteristics from images, and it is possible to not have all the necessary data for an effective categorization.

On the other hand, deep learning (DL) techniques use neural networks to automatically extract features. These systems have the capacity to learn from images and extract details that may not be immediately noticeable to human viewers. Convolutional neural networks (CNNs), U-Net, ResNet, and Masked RCNN are examples of DL models that have demonstrated notable gains in difficult biological tasks including segmentation and classification.

For instance, radioactive material was divided into several categories and nuclear sites were found using a multi-scale deep residual aggregation network [22]. Clustered nuclei were divided using the Feature Pyramid Network (FPN) [23] and U-Net architecture. Deep learning models [24] were used to identify nuclei outlines, and segmentation was conducted using an iterative region expanding technique.

Better performance in many biological activities has been made possible by the exponential expansion of DL architectures and computer vision techniques in recent years. Researchers can increase their performance in nucleus segmentation and other difficult biological image processing tasks by utilizing the characteristics of DL models.

In their research, Adu et al. [25] established a brand-new method for examining lung and colon cancer histology images termed DHSCapsNet. To improve efficiency, the network integrates DHSCaps with encoder characteristics. In particular, the convolutional layers that collect important features are where the encoder features are formed. Additionally, HSquash is used to glean important data from people with various backgrounds. A CNN Pre-Trained Diagnostic Network created in the study by Mangal et al. [26] was created particularly for the categorization of colon and lung cancer. The network analyzed histology slides using a basic CNN architecture. The network impressively attained high accuracy for detecting colon and lung cancer. The concept described by Ali and Ali [27] uses a capsule network with many sources to build a diagnostic architecture for aberrant cell cancer in the lung and colon. The convolutional layer block (CLB) and separable CLB are the two unique building components that make up the capsule network. While the separable CLB is in charge of processing histopathological images, the CLB is in charge of processing pathological images. This method uses the capsule network architecture to provide a thorough examination of various input data types. The research of Mehmood et al. [28] offers a useful framework for the precise finding of lung and colon cancer cells. The four key levels of the network were modified by the researchers using the AlexNet design. They achieved a noteworthy accuracy of 89% by fine-tuning the model using the changed layers and training it on a dataset. This method highlights the possibility of modifying current deep learning architectures for enhancing lung and colon cancer diagnosis. Toğaçar [29] established the DarkNet-19 model as the foundation for training a lung and colon cancer-specific dataset. By learning the DarkNet-19 architecture from scratch as opposed to utilizing pre-trained models, the researchers were able to teach it the specific features of the cancer dataset. They used the equilibrium method, which assisted in locating and choosing the most pertinent characteristics for the classification job, to increase the model’s efficacy. The system distinguished between ineffective traits and effective ones, giving greater weight to the latter. After that, a support vector machine (SVM) was utilized to categorize the lung and colon cancer samples using the chosen effective characteristics. The objective of this strategy was to ameliorate the model’s accomplishment and accuracy by concentrating on its most illuminating and discriminative properties. The work by Masud et al. [30] introduces a deep learning model for the categorization of five different types of lung and colon cancers. To amend the quality of the input images and guarantee the best possible performance of the model, the researchers used optimization techniques for them. Both 2D Fourier and 2D wavelet features, which are frequently used methods for analyzing signals and images, were recovered from the images in order to extract pertinent information. The authors produced a deep learning model that has a high accuracy by using these characteristics. This demonstrates how well their suggested method works for correctly identifying and categorizing the various forms of colon and lung cancer. In their work, Hamida et al. [31] used histological images from the AiCOLO dataset to categorize areas afflicted by colon cancer using four pre-trained deep learning architectures. They used SegNet and UNet for pixel segmentation operations, which allowed them to precisely identify the impacted areas. Additionally, they used a pre-trained ResNet model, which successfully classified the areas of colon cancer with an excellent accuracy. This study illustrates the effectiveness of using pre-trained architectures and deep learning architectures for the precise analysis and categorization of colon cancer in histological images. In order to detect aberrant cells and assess biomarkers for the diagnosis of colon cancer, Sarker et al. [32] introduced a deep learning strategy to handle segmentation issues. The suggested methodology accurately identifies and highlights aberrant cells using cutting-edge image segmentation techniques. With the help of these segmentation capabilities, it is possible to not only spot probable problem regions but also provide annotations that are very helpful to doctors throughout the diagnostic procedure. This research makes use of deep learning algorithms to create cutting-edge tools that assist medical practitioners in accurately diagnosing colon cancer and measuring pertinent biomarkers. A pretrained architecture, such as ResNet, for identifying colon tumor was introduced by Sarwinda et al. [33]. On two datasets that were split into 20% and 40% subsets, the model’s performance was assessed. ResNet50 fared better than ResNet18 among the studied models, obtaining a sensitivity of 87% and an accuracy of 80%. A technique was developed by Zhou et al. [34] to categorize whole slide imaging (WSI) images using labeled data. By incorporating characteristics from various magnification levels, the network graded colorectal cancer with an accuracy of 94.6%. A 1D CNN network was developed by Moitra and Mandal [35] to classify small cell lung tumors. To outperform more conventional machine learning methods, the network combined clinical characteristics with hybrid features from images.

An AI-based pre-screening tool that can distinguish between normal and malignant colon samples was developed by [36] with the goal of assisting pathologists throughout the diagnosing procedure. With just slide-level labels needed, the program uses weakly supervised deep learning to extract histological patterns from complete slide images. It demonstrated great accuracy in cross-validation and external validation, and it may be useful for clinical settings to help with colorectal biopsy pre-screening. The understanding of the model’s forecasting and the connection between neoplastic histology and genetic heterogeneity was improved through genetic analysis and route investigation.

The necessity to distinguish between benign and malignant colorectal adenomas, which are precursors to colorectal cancer, is addressed in this study. The suggested method, known as MIST, makes use of a multiple instance learning network based on the Swin Transformer and is capable of correctly classifying whole slide images (WSIs) of colorectal adenomas using just slide-level labels. The model demonstrated a high accuracy in external validation and was trained and validated on a dataset of 666 WSIs from patients with colorectal adenoma. The results of the interpretability study are congruent with the local pathologists’ areas of interest. MIST offers a viable and practical approach to colorectal cancer screening, supporting physicians’ judgment and perhaps lowering CRC patient mortality [37].

The domain shift issue that arises in machine learning models when training and testing data have distinct distributions and varying color intensities, addressed by [38]. The authors suggest a methodology to address this problem that combines stain normalization methods with a collection of precise, scalable, and reliable convolutional neural networks (CNNs) termed ConvNexts. The improvement brought about by combining five widely used stain normalization approaches is empirically investigated in the study. On three datasets including more than 10,000 images of colon histopathology, the suggested method’s classification performance is assessed.

This study focuses on Invasive Ductal Carcinoma Breast Cancer (IDC-BC), a common and often asymptomatic cancer type with high mortality rates. The research explores the potential of pre-trained convolutional neural networks (CNNs), including EfficientNetV2L, ResNet152V2, and DenseNet201, either individually or as an ensemble, for IDC-BC grade classification using the DataBiox dataset. Data augmentation is used to address data scarcity and imbalances. The proposed ensemble model outperforms existing state-of-the-art techniques, achieving a 94% classification accuracy and significant area under the ROC curves for grades 1, 2, and 3 (96%, 94%, and 96%, respectively) in the Databiox dataset [39].

The goal of this work was to create a computer-aided diagnostic (CAD) methodology that could automatically categorize lung tissue histopathology images. The CAD system was created and validated using two datasets: a private dataset and a public dataset. The public dataset comprised 15,000 images categorized into three groups, whereas the secret dataset contained 94 images divided into five categories. Machine learning was used to classify the images, along with traditional texture analysis (TA) and homology-based image processing (HI), two different methods of extracting image features. In all datasets, the CAD methodology with HI performed higher-up than the one with TA, proving the use of HI for precise lung tissue categorization [40].

Bhattacharya et al. [41] employs computer vision algorithms to identify cancer, especially lung and colon carcinomas. The paper offers a system that combines two deep learning models (ResNet-18 and EfficientNet-b4-widese) with AdBet-WOA, a hybrid meta-heuristic optimization technique. Deep features are retrieved when the deep learning networks are learned on the LC25000 dataset. The suggested technique uses a support vector machine (SVM) classifier to accurately classify lung cancer, colon cancer, and a combination of the two. The data demonstrate nearly flawless precision for colon, lung cancer, and combination categorization. The suggested method performs better in terms of feature reduction and classification performance than existing optimization techniques.

Diao et al. [42] presents a unique method for the accurate categorization of histopathology images termed deep multi-magnification similarity learning (DSML). The method focuses on the largely unexplored fusing of cone-shaped histopathological images at various magnifications. The difficulty of comprehending cross-magnification information transmission is solved by DSML, which also makes it simple to visualize feature representations. A similarity cross-entropy loss function is used to determine how similar bits of information are at different magnifications. Experiments were conducted using clinical nasopharyngeal carcinoma and public breast cancer datasets to demonstrate how well the DSML performed in terms of categorization when compared to other comparable methodologies. The report also discusses the reason behind the efficacy of multi-magnification approaches.

Due to the similarities in the early stages of lung and colon cancer tumors, the researchers intended to produce encouraging findings in the detection of both diseases. The key goal for researchers in this area continues to be promising accuracy. The goal of this work was to represent each class with individual properties by extracting characteristics from deep learning architectures and merging them. In order to obtain promising results, it also mixed handmade features with characteristics from deep learning architectures.

## 3. Data and Materials

This section describes the datasets that were used in this study. The repository is composed of colon and lung cancer histopathological medical samples.

### 3.1. Dataset

**Figure 1:**
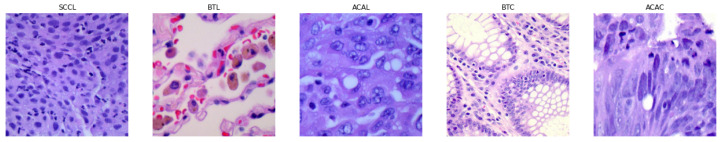
A defined number of histological image samples are included in the LC25000 dataset.

The LC25000 dataset, which consists of histological images of lung and colon cancer, was used in this study. It was downloaded from the publicly accessible Kaggle website. A dataset of 25,000 images segregated into two forms of colon tumor and three classes of lung tumor was assembled by Andrew Borkowski and colleagues at James Hospital Tampa, Florida. A balanced dataset is produced by evenly distributing 5000 images across each kind. The kinds are ACAL (adenocarcinoma of lung), BTL (benign tissue of lung), and SCCL (squamous cell carcinoma of lung) for lung cancer. For colon cancer, the types are ACAC (adenocarcinoma of colon) and BTC (benign tissue of colon). While lung adenocarcinoma and squamous cell carcinoma are the most common types of lung cancer, colon adenocarcinoma accounts for the majority of instances of colon cancer. The dataset also contains samples of benign tissue. The five classes’ samples from the LC25000 dataset are shown in Figure 1.

### 3.2. Stain Normalization

To standardize color and intensity differences in histopathology images of colon and lung cancer, a procedure called stain normalization is utilized. To improve visibility, dyes are used to stain these images; however, different staining methods may lead to variable color and intensity levels. These variances are adjusted by stain normalization, which creates a uniform and similar images. The method increases the accuracy of machine learning models and assures dependable and consistent interpretation of the images by minimizing the influence of staining discrepancies.

(1) $$\begin{array}{cc} L,A,B & =F(R,G,B)\\ NB & =NB_{F}\left(A,B\right)\\ SM & =SVD(NB)\\ OD & =FOD(L,A,B)\\ SC & =SM\times OD\\ R_{n},G_{n},B_{n} & =F^{\prime }\left(L,A,B\right)\\ R_{n} & =R\times \left(\frac{SN[0]}{SN_{r}\left[0\right]}\right)\\ G_{n} & =G\times \left(\frac{SN[1]}{SN_{r}\left[1\right]}\right)\\ B_{n} & =B\times \left(\frac{SN[2]}{SN_{r}\left[2\right]}\right) \end{array}$$

**Figure 2:**
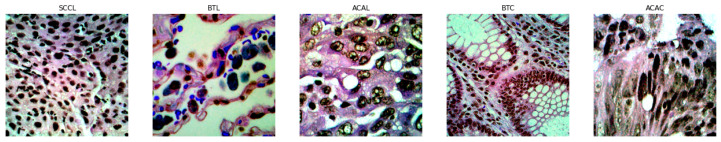
In this research, the Macenko method is utilized for stain normalization. Non-background samples are utilized to calculate the stain vector using singular value decomposition (SVD).

Stain normalization depicted in Equation (1) is used while histopathology images come from several laboratories using different acquisition techniques, which causes inconsistencies and abnormalities in color saturation. Before using the complete dataset for deep learning models, normalization is necessary to ensure consistency and eliminate noise. Figure 2 shows the results of the stain normalization procedure. Prior to applying the Macenko technique, the RGB to LAB color space conversion of the input image is necessary. The L (lightness), A (green-red axis), and B (blue-yellow axis) channels are separated from the image during this conversion. In the Macenko approach, a set of non-background pixels in the A and B channels are used to generate the stain matrix. A threshold or other segmentation methods are often used to generate these non-background pixels. Singular value decomposition (SVD) is utilized to calculate the stain matrix on the chosen pixels in order to estimate the stain vectors. The Macenko technique depicted in Figure 2 determines the stain concentrations for each pixel in the image after obtaining the stain matrix. The pixel values must be changed from the LAB color space to the OD (optical density) space to do this. Stain normalization is achieved using the predicted stain concentrations in the last step. The stain concentrations are used to modify the color values of each pixel to match a chosen standard or reference image after the source sample is regenerate from the LAB color space back to the RGB color space.

Due to its efficiency and adaptability to different staining variances, the Macenko technique for stain normalization in histopathological images offers special benefits. Stain variation between various tissue samples is a problem that is frequently present in histopathological images due to variations in staining techniques, laboratory settings, and tissue preparation. The Macenko method is a widely used stain normalization approach that solves this issue.

A dataset with sparse colon and lung cancer annotations is segmented using pre-trained Mask R-CNN models, which are renowned for their deep segmentation skills. We seek to learn about and comprehend the shape of the colon and lung cancer tissues in the dataset by utilizing these models. The datasets that are accessible contain few annotations, and pathologists must spend time and be prone to mistakes manually annotating them.

**Figure 3:**
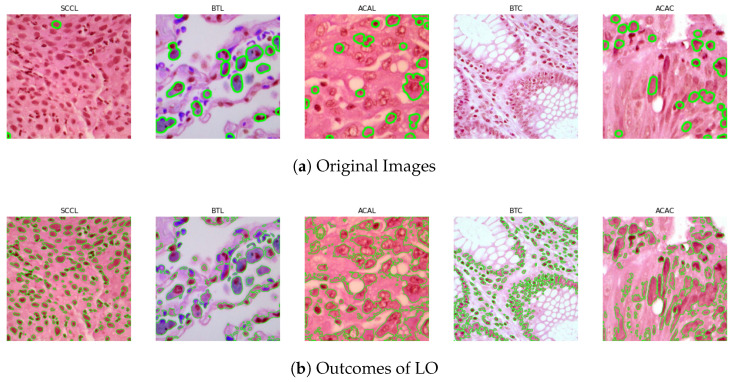
The label optimizer algorithm was employed to accurately label mitotic cells that may have been overlooked by the MASK-RCNN method. By comparing the results obtained from MASK-RCNN and the label optimizer, we observed the effectiveness of our proposed algorithm in improving the labeling of these specific cellular structures.

We implemented a label optimizer based on ResNet and Mask-RCNN architecture to address the issue of manual annotations in the datasets.The entire process of improving images with weak labels is illustrated in [43]. To create tumor cell bounding boxes, a region proposal network (RPN) was given the appropriate characteristics that ResNet had extracted from the updated dataset. To create a mask, coordinates, and classification information for the tumor cells, these extracted features and bounding boxes were then sent through another RPN made up of a feature pyramid network and fully connected layers. The collected information was then used to produce masks for false positives or annotate the weakly labeled tumor cells in our proposed label optimizer (LO), as illustrated in [43]. To enhance the weakly labeled dataset’s quality, we used MASK-RCNN. We were able to hone the poorly tagged images and obtain successful outcomes as depicted in Figure 3 by utilizing the key elements of MASK-RCNN, including area extraction, CNN feature computation using feature pyramid network (FPN), and pixel-level region classification. Our suggested CNN-based model for automated feature extraction as well as the global–local pyramid pattern (GLBP) for manual feature extraction were both then fed the optimized findings. We sought to reduce any limits or drawbacks of the CNN technique by merging these manual elements with the suggested CNN model.

We have examined the spatial patterns of pixel intensities within a local neighborhood in medical photographs using a cutting-edge method known as global–local pyramid pattern (GLPP) [43]. An image processing method called the global–local pyramid pattern (GLPP) is used to identify important characteristics in several medical image modalities. In order to examine the intensity values of pixels and their surrounding neighbors, it rotates both clockwise and anticlockwise. The steps in the GLPP process are as follows:

1. Local Neighborhood and *p* Value: Each pixel has a local neighborhood defined around it, and the “*p*” value denotes how many of the neighboring pixels were taken into account during the analysis. After each iteration, we change the value of P to extract features from local to global regions.
2. Binary Code Assignment: The assignment of a binary code to each pixel is performed by comparing its intensity value to the values of the pixels around it. A value of 1 is assigned if the intensity of an adjacent pixel is greater than or equal to the intensity of the center pixel; otherwise, a value of 0 is provided.
3. Decimal Number Translation: By repeating this process counterclockwise, the binary codes collected from the nearby pixels are converted into decimal numbers.
4. GLPP image Generation: Using the higher decimal value obtained in the previous phase, the GLPP image is generated.

**Figure 4:**
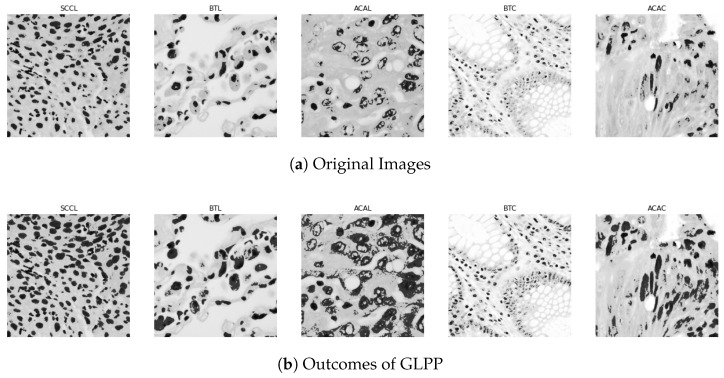
The heatmap analyzes the image and identifies specific regions where features are extracted for further analysis. It visually represents the extracted features from various of colon and lung tumor cells.

The central value, the size of the local neighborhood (neighbor), and the number of surrounding pixels (P) all have a significant impact on the properties that the GLPP extracts. To receive relevant information, these parameters must be given when using the GLPP on medical images. The results of the GLPP are shown in Figure 4, which perhaps illustrates how the method catches key structures and patterns in the medical image data.

The global–local pyramid pattern can be mathematically represented using Equation (2).

(2) $$G_{i}=f_{g}\left(I,\theta_{g}^{\left(i\right)}\right)+f_{l}\left(I,\theta_{l}^{\left(i\right)}\right)$$

where G${}_{i}$ is the feature map at the i-th scale, f${}_{g}$ is the global convolution operation, f${}_{l}$ is the local convolution operation, *I* is the source image, and $\theta_{g}^{\left(i\right)}$ and $\theta_{l}^{\left(i\right)}$ are the sets of learnable parameters for the global and local convolutions, respectively.

**Figure 5:**
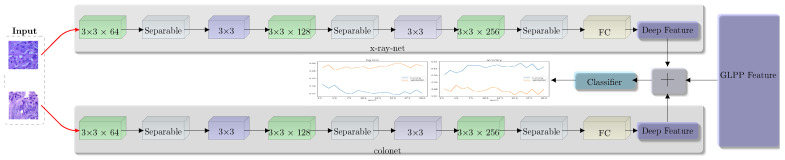
Feature extraction from different histopathological images of colon and lung for classification.

Pretrained models for categorization problems have become increasingly popular and use deep learning architectures like VGG, ResNet, DenseNet, Inception, and Xception. These designs have layers of encoding and decoding that make it easier to extract and reconstruct features. Convolutional, max pooling, and ReLU layers are often included in the encoding phase, whereas transposed convolutional layers and max pooling are used in the decoding phase for feature up-sampling. In this article, we suggest a ResNet-inspired deep residual CNN model for classifying colon and lung cancer. This model, referred to as ColonNet, is depicted in Figure 5. The architectural details of ColonNet are explained in Section 4 of this study.

## 4. Proposed Methodology

In our proposed ColonNet, we leverage the widely adopted depth-wise separable convolutions, which have proven to be efficient in various neural network architectures. The primary objective of integrating this layer into our examination is to identify local features within low magnification images. This layer divides the conventional convolution into two distinct layers. Firstly, it applies a lightweight convolutional kernel individually to each input channel, and secondly, it employs a 1 × 1 convolution for point-wise operations, generating new features. By utilizing this approach, we significantly bring down computation time in comparison to traditional layers.

**Figure 6:**
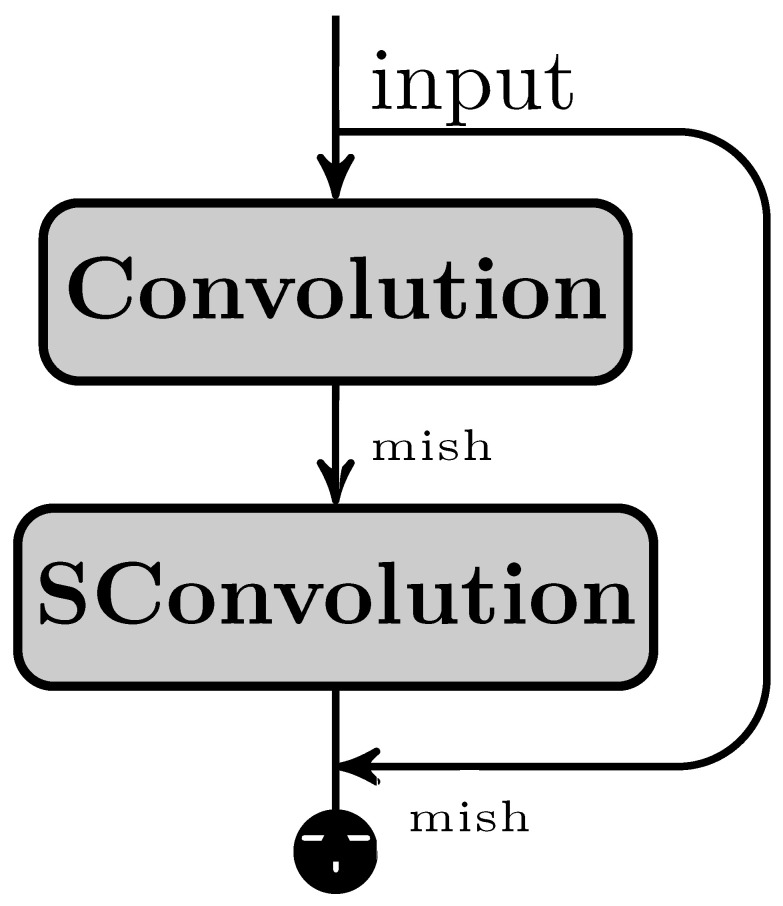
Customized skip connection-enabled deep residual network layer.

Consider an image with dimensions (X × Y × C), where C represents the number of channels. In the case of depth-wise convolution, the kernel has a size of k × k. The number of source channels is denoted as C, while the number of outcome channels is C${}_{t}$. The outcome feature map (F) resulting from traditional convolution can be seen in Equation (3), and a visual representation is provided in Figure 6. The total number of learnable parameters is determined by k × k × X × Y. On the other hand, the depth-wise convolution is illustrated in Equation (4), and the total number of learnable parameters can be computed as k × k × X + X × Y.

The symbol F represents the output feature map, and it is computed by summing over the range of ii from 0 to NN as follows:

(3) $$\begin{array}{c} F=\sum_{i=0}^{N}\left(X_{i}\cdot K_{i}^{j}+b\right) \end{array}$$

(4) $$\begin{array}{c} F=(X_{i}\cdot K_{i}+b) \end{array}$$

The combination of ResNet and depth-wise separable convolution has confirmed encouraging findings in colon and lung cancer datasets, particularly in the LC25000 dataset. In order to further enhance the performance of the convolutional neural network (CNN), we employed a technique called feature grafting (FG). The primary objective of feature grafting in CNN is to acquire relevant features that may not have been effectively extracted through simple convolution alone.

To achieve feature grafting, we utilized the global–local pyramid pattern (GLPP) to retrieve the appropriate features. This methodology retrieves features in a pyramidal format, ranging from low- to global-level features. The extraction process follows a circular updating approach, iterating through each point and obtaining the maximum features from those values. These features are then concatenated in both clockwise and counterclockwise directions, resulting in a single vector that encapsulates the combined information.

The traditional CNN methodologies are predominantly utilized in CNN training. However, to incorporate manual features within the convolution layer and enhance the classification efficacy of colon and lung cancer, we commence a new thought called the custom layer convolution. This approach involves the automated extraction of features combined with the grafting of handcrafted features.

(5) $$\begin{array}{cc} {\left(\chi *\kappa \right)}_{ij} & =\sum_{u=0}^{f_{1}-1}\sum_{v=0}^{f_{2}-1}\kappa_{u,v}\cdot \left(\left(\chi_{i+u,j+v}\right)+\left(F_{GLPP}^{T}\right)\right)+b \end{array}$$

In Equation (5), we depict the ingestion of handcrafted features $F_{GLPP}^{T}$ into the automated features. By applying backpropagation using the chain rule, we subtract the handcrafted feature prior to computing the *∂* derivative. The chain rule is used to compute gradients on individual weights. Equation (6) shows the removal of handmade characteristics, which are then combined into a single vector ($F_{GLPP}^{T}$).

(6) $$\begin{array}{cc} \frac{\partial \lambda }{\partial \omega_{u^{\prime },v^{\prime }}^{l}} & =\sum_{i=0}^{H-f_{1}}\sum_{j=0}^{\omega -f_{2}}\frac{\partial \lambda }{\partial \upsilon_{i,j}^{l}-\left(F_{GLPP}^{T}\right)}\frac{\partial \upsilon_{i,j}^{l}-\left(F_{GLPP}^{T}\right)}{\partial \omega_{u^{\prime },v^{\prime }}^{l}}\\ \; & =\sum_{i=0}^{H-f_{1}}\sum_{j=0}^{\omega -f_{2}}\delta_{i,j}^{l}\frac{\partial \upsilon_{i,j}^{l}-\left(F_{GLPP}^{T}\right)}{\partial \omega_{u^{\prime },v^{\prime }}^{l}} \end{array}$$

### 4.1. Feature Engineering

Feature engineering or grafting is a concept in convolutional neural networks (CNNs) that aims to incorporate manual or handcrafted features into the network for improved performance. In CNNs, lambda layers provide a flexible framework to introduce customized computations within the network architecture. Feature engineering takes advantage of this flexibility by using a lambda layer to merge the manually crafted features such as GLPP with the automated features obtained from the network such as ColonNet. Initially, we generate feature maps that capture relevant patterns and information from the input data via ColonNet. These features represent the network’s learned representations. Next, the lambda or custom layer is introduced to graft the GLPP features into the network. This layer acts as a bridge between the automated features and the manually crafted features. We combine the two sets of features using a concatenation approach. By incorporating the GLPP features into ColonNet through feature engineering, the CNN gains the ability to leverage both the learned representations from automated feature extraction and the domain-specific knowledge encoded in the manual features. During forward propagation as depicted in Equation (5), the lambda layer performs the defined custom operation, merging the automated and manual features into a fused representation. During backpropagation, as depicted in Equation (6), the gradients flow through the lambda layer, allowing the network to learn the optimal weights and parameters for the grafting operation. The gradients are computed based on the loss function and propagated through the network for further parameter updates. Using feature engineering, we enhance the network’s capacity to capture and exploit important information, potentially leading to improved performance on the categorization of colon and lung cancer.

### 4.2. Classification of Fused Features

We utilize the combined feature representation to train traditional machine learning classifiers such as support vector machines (SVMs), decision tree, and k-nearest neighbor (KNN). We apply the chosen classifier to the engineered features and allow it to acquire the relationship between the combined features and the corresponding class labels.

The engineered features obtained from a combination of automated and manual feature extraction methods are utilized for colon and lung cancer detection and classification. The engineered features are fed as source to three various traditional machine learning classifiers: support vector machines (SVMs), k-nearest neighbors (KNNs), and decision tree classifiers. The purpose of employing these classifiers is to leverage their classification capabilities and determine the class labels for the cells based on the fused features. Each classifier has its own approach to making predictions, with SVM using hyperplanes to separate different classes, KNN relying on the proximity of neighboring data points, and decision tree classifiers utilizing hierarchical decision rules.

To evaluate the effectiveness of the engineering manual and auto feature approach, the LC25000 public dataset is used, and data augmentation methodologies are applied to intensify the diversity and quantity of the training data. The proposed approach is then compared with baseline methods as well as state-of-the-art models to assess its performance relative to existing approaches.

## 5. Results and Discussions

In this study, the proposed approach (ColonNet) and pretrained models such as VGG, ResNet, DenseNet, Inception, and Xception are applied to histopathological images from the LC25000 dataset to diagnose lung and colon cancer and discriminate between them at early stages. The LC25000 dataset comprises 25,000 histopathological images obtained through biopsy from patients with affected tissues. The dataset is categorized into five types, including malignant and benign tumors of lung and colon cancer. The distribution of histopathological images is as follows: 5000 images of ACAC, 5000 images of BTC, 5000 images of ACAL, 5000 images of BTL, and 5000 images of SCCL. It is important to note that the dataset consists of three classes of malignant lesions and two classes of benign lesions. Additionally, all classes in the dataset are balanced, meaning they contain an equal number of histopathological images.

The results are obtained using convolutional neural networks (CNNs) when combining fusion features from our proposed approach and manual features, as well as fusion features from pretrained models and manual features. Two methodologies were developed for feature merging. Initially, the method consisted of gathering pretrained model features, lowering their dimensionality, and integrating them with GLPP features. The second procedure, like the first, included extracting ColonNet features, reducing their size, and integrating them with GLPP features. The ColonNet model is trained using the fusion task features, and during the validation process, the weights are adjusted to optimize performance. The performance of the trained ColonNet model is then evaluated on test datasets. The objective is to attain promising findings for the early recognition and discrimination of lung and colon tumors.

A key indicator of how well the model’s predictions are made overall is accuracy. Out of all the occurrences in the dataset, it determines the proportion of instances that were correctly categorized. These metrics, namely precision, recall, and F1-score, are frequently employed in classification tasks. Focusing on the accuracy of positive predictions, precision evaluates the proportion of accurate positive forecasts among all positive predictions. Focusing on the model’s capacity to accurately identify positive cases, recall (also known as sensitivity) measures the percentage of true positive predictions out of all real positive instances. The model’s performance on both positive and negative classes is balanced by the F1-score, which is the harmonic mean of precision and recall. The model’s performance in classification tasks is assessed using AUC-ROC. The confusion matrix presents the model’s predictions in tabular form, emphasizing the proportion of true positive, true negative, false positive, and false negative occurrences.

The accuracy graph compares the performance of five different pretrained CNN models such as VGG, ResNet, DenseNet, Inception, Xception, and our proposed approach (ColonNet). Each model is evaluated on a specific task, and the accuracy metric measures the percentage of correctly classified instances. VGG constantly exhibits a high level of accuracy over the whole dataset, demonstrating how successful it is in classifying data. Out of the five models, it has the highest accuracy, averaging about 89.07. The VGG model successfully extracts the important information from the histology images and distinguishes between distinct classes to classify colon and lung tumors with excellent accuracy. ResNet works admirably as well, with a high accuracy that is comparable to VGG. The robustness of its categorization is demonstrated by the fact that it maintains a constant degree of accuracy over the whole dataset. ResNet displays great accuracy in classifying colon and lung cancer by successfully identifying complex patterns and characteristics in the histology images. With the help of its deep layers, it can learn complicated representations, which enhances classification performance. ResNet and DenseNet both perform admirably, with excellent accuracy that is consistent across the dataset. Although significantly less accurate than ResNet and VGG, it nevertheless has solid classification skills. Due to its extensive interconnectedness, which enhances information flow via the network, DenseNet works well. In comparison to VGG, ResNet, and DenseNet, Inception achieves a comparatively lower accuracy. However, it shows a significant improvement in accuracy compared to the baseline. The graph indicates a consistent increase in accuracy as the dataset progresses. The Inception model exhibits great performance in the categorization of colon and lung cancer by successfully collecting both local and global characteristics from the histological images. Xception performs slightly better than Inception but lags behind VGG, ResNet, and DenseNet in terms of accuracy. The graph shows a gradual increase in accuracy over the dataset, indicating the model’s ability to learn and improve its classification performance.

Our proposed model ColonNet outperforms the five pretrained CNN models (VGG, ResNet, DenseNet, Inception, and Xception) in terms of accuracy. It is specifically designed and trained for the task of colon and lung cancer classification. It incorporates unique architectural elements and training techniques that contribute to its superior performance. One key aspect of ColonNet is its ability to effectively capture and utilize both local and global features present in colon and lung histological images. It employs specialized convolutional layers, such as depth-wise separable convolutions, which enhance the extraction of relevant features from the images. Depth-wise Separable Convolutions, a widely used method renowned for its effectiveness in a variety of neural network configurations, are a key component of ColonNet. In contrast to typical layers, this layer divides the usual convolution process into two different stages, speeding up computing. This method’s application in the context of cancer diagnosis is innovative, enabling ColonNet to rapidly find local characteristics in images with low magnification.This enables ColonNet to capture intricate patterns and subtle details that are crucial for accurate cancer classification. Additionally, ColonNet incorporates feature grafting, a novel concept that combines automated feature extraction with handcrafted features. This integration allows the model to benefit from both the learned representations from the pretrained layers and the domain-specific knowledge encoded in the handcrafted features. This fusion of features enhances the discriminative power of the model and improves its accuracy in distinguishing between different classes of colon cancer.

Table 1 depicts the performance outcomes of various pretrained models that have been used for the classification of the LC25000 dataset. These systems are compared with the achievement of the projected system in the current study. The findings indicate that the proposed system surpasses all the systems used in previous studies. This suggests that the proposed system has achieved higher accuracy, precision, recall, or other performance metrics compared to the existing systems. The superior execution of the proposed system highlights its effectiveness and demonstrates its potential for improving the categorization accuracy of the LC25000 dataset. It indicates that the proposed system has successfully addressed some of the limitations or shortcomings of the previous systems, leading to improved classification results.

**Figure 7:**
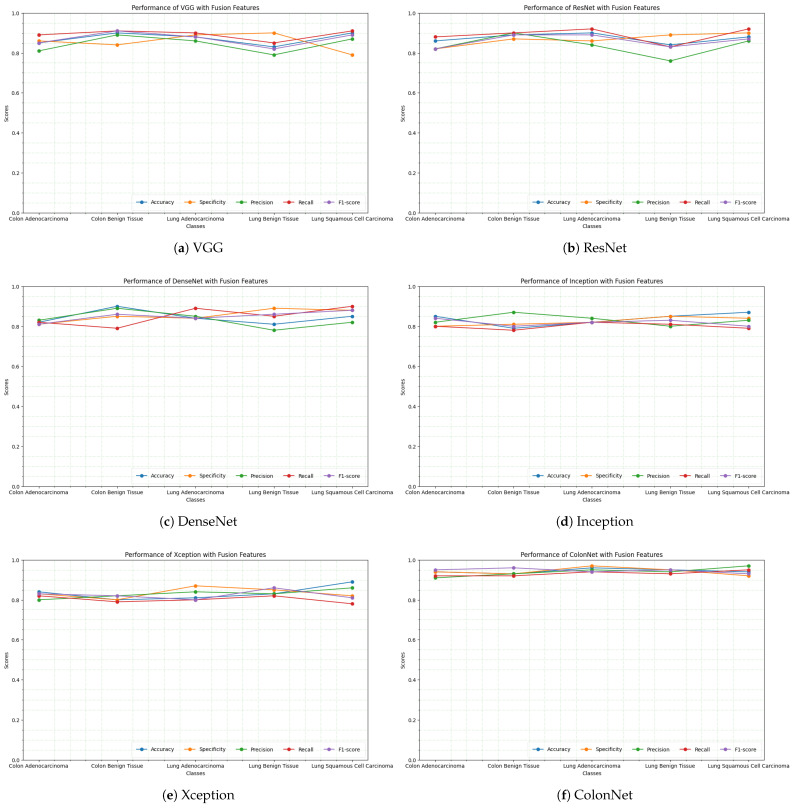
Accuracy graph of five classes (colon adenocarcinoma, colon benign tissue, lung adenocarcinoma, lung benign tissue, lung squamous cell carcinoma) using different pretrained models (VGG, ResNet, DenseNet, Inception, Xception) and our proposed approach for colon and lung classification.

**Figure 8:**
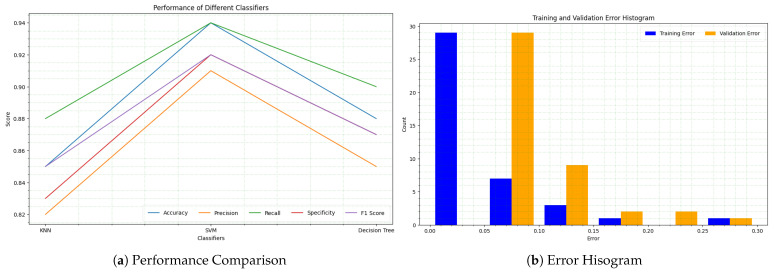
Performance comparison of different machine learning classifiers for colon and lung cancer classification.

The utilization of the GLPP pad with minimum sub-vector and boundary values yields promising outcomes and exhibits enhanced performance regarding F1 score, precision, and recall. Precision and sensitivity play a critical role, particularly in datasets with imbalanced classes. By effectively identifying positive predicted mitotic cells while minimizing false positives, the GLPP pad significantly improves precision and sensitivity. This achievement carries significant implications for patient prognosis, treatment, and assessment, as the precise identification of mitotic cells is crucial for accurate diagnosis and informed decision-making. Based on the precision-based study, the proposed model ColonNet demonstrates a precision of 96.11, effectively distinguishing genuine positive cases belonging to the tumor class. It outperforms the top performing base model by 11% and the state-of-the-art CNN by 15%. The comparison in Table 1 shows that ColonNet significantly reduces the misclassification of lesion cells compared to the base classifiers and state-of-the-art CNNs. Figure 7 provides an illustration of challenging benign samples that are misclassified by the basic classifiers but accurately classified by ColonNet. The precision and ROC curves for ColonNet and existing CNNs on the dataset are depicted in Figure 7. In contrast, the precision curve focuses on the minority class, showcasing the model’s precision and detection rate. In order to address this issue, a precise and sophisticated categorization system is necessary to exclude them. With this in mind, we have meticulously developed a CNN-based model that can effectively capture the different patterns of mitosis across different stages, taking into account the heterogeneity of the data. We included principles like area homogeneity and in-variance, asymmetric split transform merge, dilated convolution, attention mechanisms, and residual learning in our CNN design. Through Figure 8 and Table 1, we have demonstrated that our proposed model achieves significant diversification and performance improvements.

Using the five different pretrained models (VGG, ResNet, DenseNet, Inception, and Xception) and our proposed approach (ColonNet), these machine learning classifiers (SVM, KNN, and decision tree) can leverage the extracted features for accurate categorization of colon and lung cancer. The pretrained models and ColonNet provide a strong foundation by extracting relevant features from the histological images, and the machine learning classifiers utilize these features to make predictions. The combination of modern feature extraction methods with well-established machine learning algorithms improves the accuracy and reliability of colon and lung cancer categorization. Figure 8 displays the performance of several machine learning classifiers in the categorization of colon and lung cancer, including SVM, KNN, and decision tree. The line graph depicts the accuracy, specificity, precision, recall, and F1-score for each classifier. The findings illustrate the classifiers’ variable efficacy in reliably recognizing and discriminating between different cancer kinds. The findings shed light on these classifiers’ potential for colon and lung cancer classification tasks.

The distribution of training and validation errors for the colon and lung cancer datasets is shown by the error histogram. The histogram is split into numerous bins, each of which represents a different range of error values. The error values are represented on the x-axis of the histogram, while the count of occurrences inside each bin is represented on the y-axis. The histogram in the instance of the colon and lung cancer dataset displays the distribution of errors for different classifiers. It assists us in understanding the variability in classifier performance and identifying any potential flaws or areas for development. We can examine the efficacy of the classifiers in properly predicting the cancer classifications and establish the overall performance of the classification models by analyzing the histogram.

By displaying the distribution of predicted class labels vs genuine class labels, the confusion matrix gives useful insights into the performance of the five pretrained CNN models (VGG, ResNet, DenseNet, Inception, and Xception).

**Figure 9:**
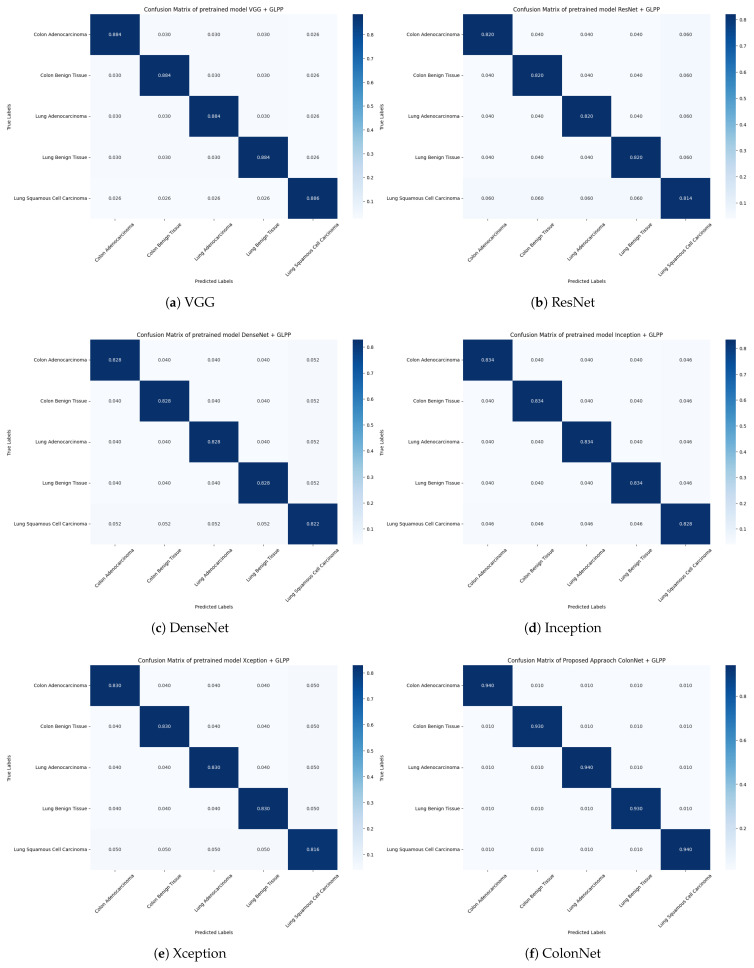
Confusion matrix of different pretrained models (VGG, ResNet, DenseNet, Inception, and Xception) and our proposed approach for colon and lung classification.

The confusion matrix shown in Figure 9 offers a thorough summary of the model’s performance across all classes, revealing both its strengths and shortcomings. It enables us to measure the model’s overall accuracy as well as its performance on particular classes. We can assess which model has superior overall classification performance and find any changes or trends in misclassifications across various classes by comparing the confusion matrices of the different pretrained CNN models (VGG, ResNet, DenseNet, Inception, and Xception).

The confusion matrix for pretrained models depicts the distribution of expected vs. true class labels. Each member in the matrix indicates the number or percentage of cases that were categorized as belonging to a certain true class label while being classed as belonging to a specific anticipated class label. We can tell how well VGG did in appropriately categorizing examples across different classes by inspecting the values in the matrix. We can determine which classes were correctly predicted and which had greater misclassification rates.

The ROC curves for the five pretrained CNN models provide insights into their performance in multi-class classification tasks. The ROC curve is a graphical representation of the true positive rate (sensitivity) against the false positive rate (1-specificity) at various classification thresholds. It illustrates the trade-off between sensitivity and specificity for different decision thresholds of the model’s predicted scores. The VGG model shows a moderate performance with an AUC score of 0.87. It achieves a balance between sensitivity and specificity across different thresholds. The pretrained model DenseNet exhibits slightly lower performance compared to VGG, as indicated by its AUC score of 0.82. It demonstrates discrimination between positive and negative samples. The ResNet performs even better with an AUC score of 0.81 compared to DenseNet. Its ROC curve indicates a true positive rate for most false positive rates, suggesting good overall performance.

**Figure 10:**
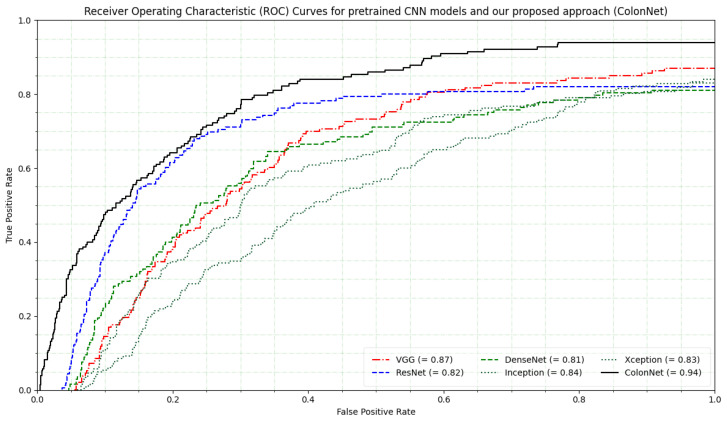
Receiver operating characteristic (ROC) curves for pretrained CNN models such as VGG, ResNet, DenseNet, Inception, Xception and our proposed approach (ColonNet).

Inception shows acceptable performance among the pretrained models, with an AUC score of 0.84. Xception performs similarly to Inception and ResNet, with a comparable AUC score of 0.83. Our proposed approach ColonNet achieves the best performance among the pretrained CNN models, as reflected by its highest AUC score of 0.94. Its ROC curve is closest to the top-left corner, indicating excellent discrimination and superior classification accuracy. The curves depicted in Figure 10 illustrate the classifier’s effectiveness at different thresholds. However, in the case of an imbalanced dataset with varying costs of misclassification, the ROC curve may yield unreliable results.

## 6. Summary

This study shows that the proposed ColonNet model outperforms existing pre-trained CNN models in terms of performance when combined with depth-wise separable convolutions and GLPP feature grafting. This development implies that combining these methodologies can produce a more precise and reliable categorization of cancer, particularly in difficult and complex cases. The manual feature extraction procedure effectively locates local features in low magnification images by utilizing depth-wise separable convolutions. This makes it feasible for real-time applications and huge datasets by enabling faster calculation and lowering the computational strain. The model’s capacity to correctly detect positive cases (malignant instances) and negative cases (non-cancerous instances) is demonstrated by the findings, which show high sensitivity and specificity. This capacity is essential for lowering false negatives and positives and increasing the accuracy of cancer detection. GLPP and feature grafting are utilized, which helps to extract important characteristics that may not be adequately captured by straightforward convolution alone. The methodology improves the model’s capacity to gather crucial data for precise categorization by combining domain-specific knowledge contained in manual features with learned representations via automated feature extraction. This study’s conclusions have a big impact on how doctors diagnose cancer in clinical situations. With its high accuracy and reliable performance, the proposed methodology has the potential to be included into CAD systems, aiding pathologists in making more accurate and effective diagnostic judgments. The technique’s efficacy in categorizing histopathological images of the colon and lungs shows that it has the potential for wider uses in cancer diagnosis. This method can be applied to tasks involving medical imaging and various cancer kinds, further advancing AI-driven medical diagnosis. The timely and appropriate use of therapy interventions can have a major impact on patient outcomes when malignant tumors are accurately detected in a timely manner. The improved performance of the suggested methodology offers prospective advantages in early cancer detection, resulting in more efficient therapies and possibly lowering mortality rates.

## 7. Conclusions

Lung and colon cancer are prevalent and life-threatening diseases, but early detection significantly improves the chances of survival. This study aims to enhance early detection of lung and colon cancer through the development of two strategies, each comprising two systems. The first strategy involves diagnosing the LC25000 dataset using convolutional neural networks (CNNs) with features extracted from pretrained models, specifically VGG, ResNet, DenseNet, Inception, and Xception. By leveraging the complementary strengths of these models, the system aims to enhance diagnostic accuracy. The second strategy employs our custom model to diagnose the LC25000 dataset using fusion features derived from the ColonNet model and manual features. This fusion approach aims to capture a more comprehensive representation of the dataset, leveraging both automated and manual feature extraction techniques. The suggested systems perform well in the initial identification of LC25000 dataset images. Specifically, when utilizing the fusion features of ColonNet and handcrafted features, the CNN achieves a sensitivity of 95.67%, precision of 96.11%, accuracy of 96.31%, specificity of 94.97%, and an area under the curve (AUC) of 94.71%. These results underscore the effectiveness of the proposed strategies in improving the accuracy and reliability of early cancer diagnosis using the LC25000 dataset.

## Figures and Tables

**Table 1:** Training accuracy, F1-score, specificity, precision, and sensitivity on colon and lung histopathological images using our proposed approach and different pretrained CNN models such as VGG, ResNet, Inception, Xception, and DenseNet.

Model	Sensitivity	Specificity	Precision	Accuracy	F1-Score
ResNet	87.15	86.12	86.37	86.12	87.46
VGG	85.15	86.17	86.06	89.07	84.21
Inception	85.04	81.65	82.11	83.38	84.23
Xception	81.37	82.16	83.51	84.57	82.12
DenseNet	82.05	82.63	83.13	84.37	83.67
ColonNet	87.34	85.64	84.31	86.36	84.36
ColonNet + GLPP	95.67	94.97	96.11	96.31	94.86

## Data Availability

The data that support the findings of this study are available from the corresponding author upon reasonable request.
